# Bam-readcount - rapid generation of basepair-resolution sequence metrics

**DOI:** 10.21105/joss.03722

**Published:** 2022-01-29

**Authors:** Ajay Khanna, David E. Larson, Sridhar Nonavinkere Srivatsan, Matthew Mosior, Travis E. Abbott, Susanna Kiwala, Timothy J. Ley, Eric J. Duncavage, Matthew J. Walter, Jason R. Walker, Obi L. Griffith, Malachi Griffith, Christopher A. Miller

**Affiliations:** 1 Division of Oncology, Department of Internal Medicine, Washington University School of Medicine, St. Louis, MO; 2 McDonnell Genome Institute, Washington University School of Medicine, St. Louis, MO; 3 Current Affiliation: Benson Hill, Inc. St. Louis, MO; 4 Current Affiliation: Moffitt Cancer Center, Tampa, FL; 5 Current Affiliation: Google, Inc. Mountain View, CA; 6 Siteman Cancer Center, Washington University School of Medicine, St. Louis, MO; 7 Department of Pathology, Washington University School of Medicine, St. Louis, MO; 8 Department of Genetics, Washington University School of Medicine, St. Louis, MO

## Abstract

Bam-readcount is a utility for generating low-level information about sequencing data at specific nucleotide positions. Originally designed to help filter genomic mutation calls, the metrics it outputs are useful as input for variant detection tools and for resolving ambiguity between variant callers (Koboldt et al., 2013a; Kothen-Hill et al., 2018). In addition, it has found broad applicability in diverse fields including tumor evolution, single-cell genomics, climate change ecology, and tracking community spread of SARS-CoV-2 (Miller et al., 2018; Müller et al., 2018; Paiva et al., 2020; Sun et al., 2020).

## Statement of need

Bam-readcount is designed to meet two related needs related to genomic sequence analysis. The first is rapid genotyping of specific locations from a bam file, reporting not just the dominant bases, but counts of all bases. One context in which this is important is residual disease monitoring, where base changes with frequency below the sensitivity of standard genomic variant callers may still be informative. The second is reporting 15 key metrics for each reported base, including summarized mapping and base qualities, strandedness information, mismatch counts, and position within the reads. This information can be useful in a large number of contexts, with one frequent application being variant filtering, to remove false-positive calls, either with straightforward application of heuristic cutoffs or with semi-automated machine-learning approaches (Ainscough et al., 2018; Koboldt et al., 2013b). Another common use case is in ensemble variant calling situations where there is disagreement about base counts or key metrics at particular sites. Bam-readcount can be used to produce consistent, tool-agnostic metrics that are helpful in resolving such ambiguity (Anzar et al., 2019; Kockan et al., 2017; Kothen-Hill et al., 2018).

## Implementation and results

**Figure 1:**
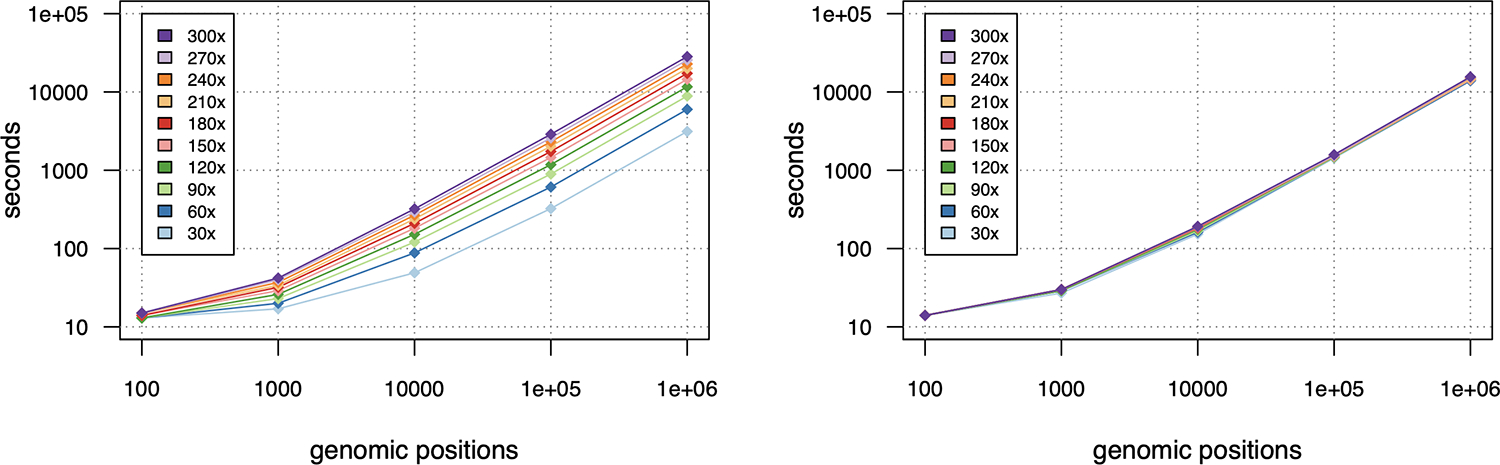
Performance of Bam-readcount when querying randomly selected genomic positions from BAMs (left) or corresponding CRAMs (right) of varying sequencing depth. Colors correspond to average sequencing depth of the downsampled BAM/CRAM file.

The ongoing adoption of compressed data formats has necessitated additions to the code, and the version 1.0 release that we report on here utilizes an updated version of HTSlib to support rapid CRAM file access (Bonfield et al., 2021). This has also improved performance, and Bam-readcount can report on 100,000 randomly selected sites from a 30x whole-genome sequencing (WGS) BAM in around 5 minutes (Griffith, Miller, et al., 2015). Its performance scales nearly linearly with the number of genomic sites queried and average sequencing depth (Figure 1). Querying the same 100,000 sites from a BAM with 300x WGS takes 48 minutes, roughly 10x as long.

Memory usage likewise is dependent on depth of sequencing, but still requires less than 1 GB of RAM for a 300x WGS BAM. Processing small CRAM files is somewhat slower than BAMs with comparable amounts of data, due to the increased CPU usage for decompression, but as depth increases, retrieval from disk becomes the bottleneck and operations on CRAMs exceed the speed of BAM. In our testing, on a fast SSD tier of networked disks, this transition occurs at a depth of about 180x. The problem is also embarrassingly parallel, so assuming adequate disk I/O, a roughly linear increase in speed can be achieved with a scatter/gather approach.

To lower barriers to adoption, we provide docker images for containerized workflows, and have developed a python wrapper that annotates a VCF file with read counts produced from this tool, available as part of the VAtools package (http://vatools.org).

## Conclusions

Bam-readcount provides fast and accurate genomic readcounts and associated metrics, which allow it to fill a key niche in many genomic workflows. It has been adopted as a lightweight variant caller, finding known mutations in pre-leukemic phenotypes and used for detecting therapy-altering mutations from cell-free DNA (Wyatt et al., 2016; Xie et al., 2014). Viral researchers have tracked nucleotide changes across samples to understand diversity in Varicella Zoster Virus Encephalitis and to perform epidemiological surveillance in wastewater of SARS-CoV-2 (Depledge et al., 2018; Mondal et al., 2021). Those with RNA-sequencing data have found it useful for identifying allele-specific expression in cancer, or for enabling copy-number detection in single-cell RNA sequencing by retrieving allele frequencies (Cancer Genome Atlas Research Network et al., 2013; Müller et al., 2018). Its feature-rich output has also enabled deep learning approaches to variant calling and filtering (Ainscough et al., 2018; Anzar et al., 2019). In these roles, and other related ones, Bam-readcount has served as key infrastructure that supports groups of all sizes, from exploratory analyses to core facility pipelines to large multi-institution workflows (Griffith, Griffith, et al., 2015; Jensen et al., 2017; Sandmann et al., 2018). In the NCI’s Genomic Data Commons pipelines alone, its use in variant filtering means that it has been run on tens of thousands of cancer genomes.

Looking forward, we anticipate that as machine learning makes deeper inroads into genomics, the ability to extract highly informative features from large cohorts in a rapid manner will continue to make Bam-readcount useful for the next generation of genomics research.

The Bam-readcount tool is available at https://github.com/genome/bam-readcount and is shared under a MIT license to enable broad re-use.

## Data Availability

The WGS data used for benchmarking is available through dbGaP study phs000159, under sample id 452198/AML31. The summary data and scripts used to generate the figure are available at https://github.com/genome/bam-readcount/tree/joss-paper/figures. An archived snapshot of this 1.0 release is available at https://doi.org/10.5281/zenodo.5142454

## Software

- Review
- Repository
- Archive
